# Erratum for “Prognostic Potential of Copper, Zinc, Copper/Zinc Ratio, Cobalamin, and Serum Amyloid A in Cats With Panleukopenia”

**DOI:** 10.1111/jvim.70127

**Published:** 2025-06-06

Yanar KE , Baysal S , Ulaş N , Aktaş MS , Timurkan MÖ , Aydın H . Prognostic potential of copper, zinc, copper/zinc ratio, cobalamin, and serum amyloid A in cats with panleukopenia. J Vet Intern Med. 2024 May‐Jun;38(3):1535‐1541. doi: 10.1111/jvim.17077. Epub 2024 Apr 13. PMID: 38613433; PMCID: PMC1109976438613433 PMC11099764

In the above‐mentioned article, there is a labeling error in Figure 3. In the ROC plot, the x‐axis was inadvertently labeled as “specificity” instead of the correct term “1 – specificity.” This error was an unintentional typographical oversight during figure preparation. The corrected Figure 3 is included below.

**Corrected Figure 3**

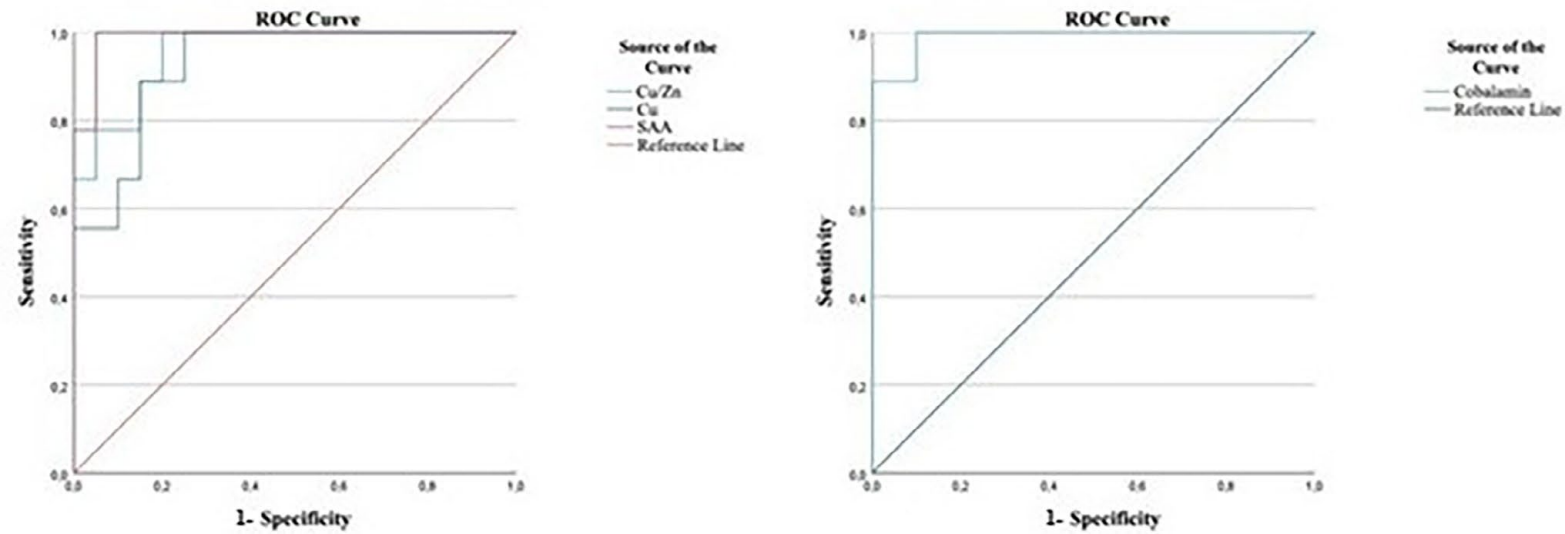

We apologize for this error.

